# Major Constituents From *Brucea javanica* and Their Pharmacological Actions

**DOI:** 10.3389/fphar.2022.853119

**Published:** 2022-03-18

**Authors:** Juan Zhang, Hong-Xi Xu, Yao-Xing Dou, Qiong-Hui Huang, Yan-Fang Xian, Zhi-Xiu Lin

**Affiliations:** ^1^ School of Chinese Medicine, Faculty of Medicine, The Chinese University of Hong Kong, Shatin, China; ^2^ School of Pharmacy, Shanghai University of Traditional Chinese Medicine, Shanghai, China; ^3^ The Second Affiliated Hospital of Guangzhou University of Chinese Medicine, Guangzhou, China; ^4^ Hong Kong Institute of Integrative Medicine, The Chinese University of Hong Kong, Shatin, China

**Keywords:** Brucea javanica, chemical constituents, pharmacological activities, molecular mechanism, anti-cancer

## Abstract

*Brucea javanica* (*Ya-dan-zi* in Chinese) is a well-known Chinese herbal medicine, which is traditionally used in Chinese medicine for the treatment of intestinal inflammation, diarrhea, malaria, and cancer. The formulation of the oil (*Brucea javanica* oil) has been widely used to treat various types of cancer. It has also been found that *B. javanica* is rich in chemical constituents, including quassinoids, triterpenes, alkaloids and flavonoids. Pharmacological studies have revealed that chemical compounds derived from *B. javanica* exhibit multiple bioactivities, such as anti-cancer, anti-bacterial, anti-diabetic, and others. This review provides a comprehensive summary on the pharmacological properties of the main chemical constituents presented in *B. javanica* and their underlying molecular mechanisms. Moreover, the review will also provide scientific references for further research and development of *B. javanica* and its chemical constituents into novel pharmaceutical products for disease management*.*

## Introduction

*Brucea javanica* (L.) Merr. belongs to the Simaroubaceae family. The medicinal use of this plant is the dry and ripe fruits, i.e., Fructus Bruceae, which is commonly called *Ya-Dan-Zi* in Chinese (Su et al., 2021). The first record of its use in Chinese medicine appeared in the book titled *The Omissions from the Compendium of Materia Medica* (*Ben-Cao-Gang-Mu-Shi-Yi*) in the Qing Dynasty (1368–1644 AD) (Ye et al., 1996; Yan et al., 2017). *B. javanica* is distributed widely throughout the tropical and subtropical zones of China, including Guangdong, Guangxi, Yunnan, and Fujian provinces. Fructus Bruceae is commonly used as medicinal herb in clinical practice in China to treat dysentery and malaria (Sakaki et al., 1986; Sornwatana et al., 2013). In addition, Fructus Bruceae is also recorded in the Chinese Pharmacopoeia for treating many diseases, including intestinal inflammation, diarrhea, malaria, and different types of cancer (Yan et al., 2017). Moreover, *B. javanica* is also useful for diseases such as abdominal pain, hyperkeratosis, hemorrhoids, and ulcers (Yoon et al., 2020). This herb is also applied topically for the treatment of warts and corns (Su et al., 2002). Furthermore, the oil of Bruceae Fructus, commonly called *Brucea javanica* oil (BJO), is a single plant-based Chinese patent medicine which contains many medicinally active constituents, including quassinoids and fatty acids. In China, two patented products of BJO, i.e., BJO emulsion injection and BJO soft capsule, have extensively been used in conjunction with chemotherapy for patients with solid tumors (Zhang et al., 2018). Its main mechanisms of action include immune boosting, anti-inflammation, and modulation of gut microbiota (Zhang et al., 2018; Su et al., 2021).

Modern pharmacological studies have also shown that the active compounds isolated from *B. javanica* possess other biological properties, including anti-viral, anti-inflammatory and cytotoxic activities (Kim et al., 2003; Dong et al., 2013; Chumkaew et al., 2017). Owing to its marked therapeutic effects, increasing number of researchers have intensively studied the chemical components of *B. javanica*. Phytochemical investigations revealed that tetracyclic triterpene quassinoids, olein, oleic acid, linoleic acid, anthraquinones, pregnane glucosides, and sesquiterpenes are the main components present in the fruits of *B. javanica*. Among these, quassinoids have been regarded as the most valuable active components (Liu et al., 2009). However, up to now, there has not been a comprehensive review on the chemical constituents and their biological activities concerning *B. javanica*. In this study, we aim to comprehensively and systematically summarize the available studies on the phytochemical and pharmacological properties, as well as their underlying mechanisms of action. Focus will be placed on the two major chemical compounds, i.e., brusatol and bruceine D. We hope that this review will provide a scientific basis for future research that may lead to better utilization of this medicinal plant.

## Methodology

The literature used for this review was sourced from electronic databases, including PubMed, Web of science, Elsevier, Google scholar, Springer, China National Knowledge Infrastructure (CNKI). Keywords such as *Brucea javanica*, Fructus Bruceae, Ya-Dan-Zi, chemical constituents, pharmacological activities, molecular mechanism were used to conduct literature search.

## Phytochemical Compounds

**FIGURE 1:**
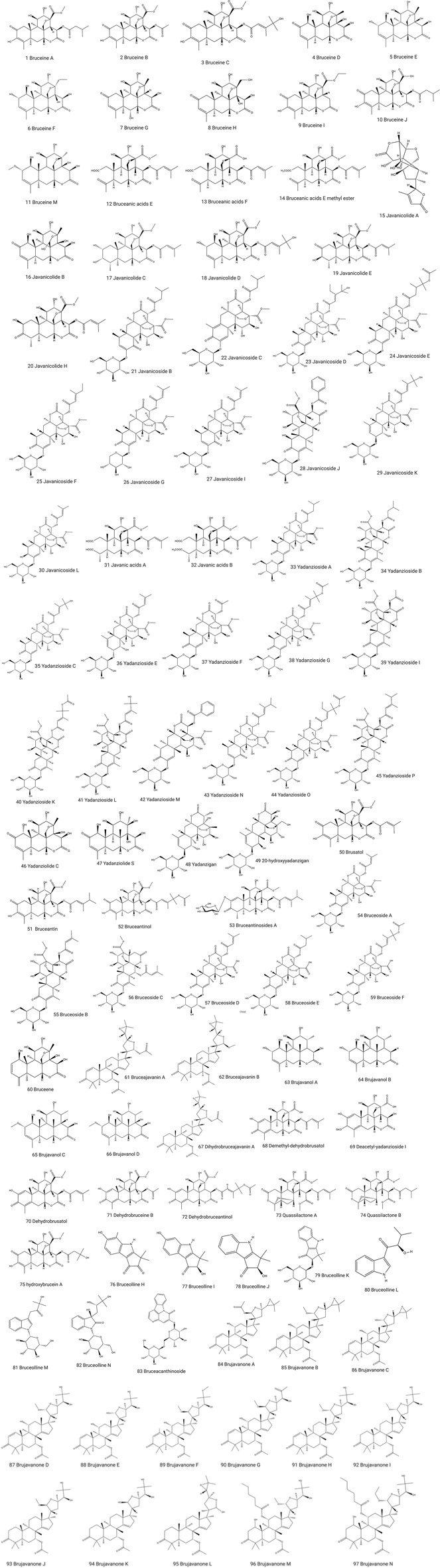
Chemical structures of the compounds isolated from *B. javanica*.

In recent decades, *B. javanica* has been subjected to intensive phytochemical investigations, and many chemical constituents, such as tetracyclic triterpene quassinoids (Chen et al., 2011; Zhao et al., 2011), olein, oleic acid, linoleic acid, anthraquinone (Chen et al., 2009), alkaloids (Liu et al., 2011) and triterpenoids (Chen et al., 2009) have been identified in this plant. Especially, tetracyclic triterpene quassinoids are the main bioactive ingredients of *B. javanica* with potent antitumor activity (Li et al., 2021). Based on the published literature, approximately 101 chemical constituents have been isolated from this plant, most of which were identified from the fruits. The main chemical constituents isolated from *B. javanica* are listed in Table 1 and Figure 1.

**TABLE 1:** The molecular formulae and source of the constituents isolated from *B. javanica*.

No	Name	Chemical formula	Extracts	Source	Ref.
Quassinoids					
1	Bruceine A	C_26_H_34_O_11_	—	Seeds	Polonsky et al. (1967)
2	Bruceine B	C_23_H_28_O_11_	—	Seeds	Polonsky et al. (1967)
3	Bruceine C	C_28_H_36_O_12_	—	Seeds	Polonsky et al. (1967)
4	Bruceine D	C_20_H_26_O_9_	EtOH	Seeds	Lee et al. (1979)
5	Bruceine E	C_20_H_28_O_9_	EtOH	Seeds	Lee et al. (1979)
6	Bruceine F	C_21_H_30_O_9_	EtOH	Seeds	Chen et al. (2013)
7	Bruceine G	C_20_H_26_O_8_	EtOH	Seeds	Duncan and Henderson, (1968)
8	Bruceine H	C_20_H_26_O_10_	EtOH	Fruits	Zhao et al. (2011)
9	Bruceine I	C_22_H_28_O_9_	—	—	Li et al. (2021)
10	Bruceine J	C_25_H_32_O_11_	EtOH	Fruits	Su et al. (2013)
11	Bruceine M	C_21_H_30_O_9_	EtOH	Fruits	Su et al. (2013)
12	Bruceanic acids E	C_25_H_32_O_12_	EtOH	Seeds	Liu J. H et al. (2012)
13	Bruceanic acids F	C_24_H_30_O_12_	EtOH	Seeds	Liu L et al. (2012)
14	Bruceanic acids E methyl ester	C_26_H_34_O_12_	EtOH	Seeds	Liu J. H et al. (2012)
15	Javanicolide A	C_26_H_34_O_11_	MeOH	Seeds	Kim et al. (2003)
16	Javanicolide B	C_20_H_26_O_10_	MeOH	Seeds	Kim et al. (2003)
17	Javanicolide C	C_26_H_36_O_11_	MeOH	Seeds	Kim et al. (2004b)
18	Javanicolide D	C_28_H_38_O_12_	MeOH	Seeds	Kim et al. (2004b)
19	Javanicolide E	C_26_H_34_O_11_	EtOH	Seeds	Liu J. H et al. (2012)
20	Javanicolide H	C_26_H_34_O_11_	EtOH	Seeds	Liu L et al. (2012)
21	Javanicoside B	C_32_H_44_O_16_	MeOH	Seeds	Kim et al. (2004b)
22	Javanicoside C	C_32_H_40_O_16_	MeOH	Seeds	Kim et al. (2004b)
23	Javanicoside D	C_35_H_48_O_17_	MeOH	Seeds	Kim et al. (2004b)
24	Javanicoside E	C_36_H_50_O_18_	MeOH	Seeds	Kim et al. (2004b)
25	Javanicoside F	C_33_H_44_O_16_	MeOH	Seeds	Kim et al. (2004b)
26	Javanicoside G	C_31_H_40_O_15_	EtOH	Seeds	He et al. (2021)
27	Javanicoside I	C_32_H_42_O_16_	MeOH	Seeds	Kim et al. (2004a)
28	Javanicoside J	C_34_H_40_O_16_	MeOH	Seeds	Kim et al. (2004a)
29	Javanicoside K	C_34_H_48_O_17_	MeOH	Seeds	Kim et al. (2004a)
30	Javanicoside L	C_32_H_46_O_16_	MeOH	Seeds	Kim et al. (2004a)
31	Javanic acids A	C_26_H_34_O_13_	EtOH	Seeds	Liu J. H et al. (2012)
32	Javanic acids B	C_27_H_36_O_13_	EtOH	Seeds	Liu L et al. (2012)
33	Yadanzioside A	C_32_H_44_O_16_	EtOH	Fruits	Su et al. (2013)
34	Yadanzioside B	C_32_H_44_O_17_	EtOH	Fruits	Sakaki et al. (1985)
35	Yadanzioside C	C_34_H_46_O_17_	EtOH	Fruits	Sakaki et al. (1985)
36	Yadanzioside E	C_32_H_44_O_16_	EtOH	Fruits	Sakaki et al. (1985)
37	Yadanzioside F	C_29_H_38_O_16_	EtOH	Fruits	Ye et al. (2015)
38	Yadanzioside G	C_36_H_48_O_18_	EtOH	Fruits	Zhao et al. (2011)
39	Yadanzioside I	C_29_H_38_O_16_	EtOH	Seeds	Yoshimura et al. (1985)
40	Yadanzioside K	C_36_H_48_O_18_	EtOH	Seeds	Sakaki et al. (1986)
41	Yadanzioside L	C_34_H_46_O_17_	EtOH	Seeds	He et al. (2021)
42	Yadanzioside M	C_34_H_40_O_16_	EtOH	Fruits	Ye et al. (2015)
43	Yadanzioside N	C_34_H_46_O_16_	MeOH	Seeds	Kim et al. (2004b)
44	Yadanzioside O	C_37_H_50_O_18_	MeOH	Seeds	Kim et al. (2004b)
45	Yadanzioside P	C_34_H_46_O_16_	MeOH	Seeds	Kim et al. (2004b)
46	Yadanziolide C	C_20_H_26_O_9_	EtOH	Seeds	He et al. (2021)
47	Yadanziolide S	C_20_H_28_O_9_	EtOH	Fruits	Su et al. (2013)
48	Yadanzigan	C_26_H_38_O_14_	MeOH	Seeds	Zhan et al. (2020)
49	20-hydroxyyadanzigan	C_26_H_38_O_15_	MeOH	Seeds	Zhan et al. (2020)
50	Brusatol	C_26_H_32_O_11_	EtOH	Seeds	Sim et al. (1968)
51	Bruceantin	C_28_H_36_O_11_	EtOH	Fruits	Su et al. (2013)
52	Bruceantinol	C_30_H_38_O_13_	EtOH	Seeds	Kupchan et al. (1975)
53	Bruceantinosides A	C_34_H_46_O_16_	EtOH	Fruits	Zhao et al. (2011)
54	Bruceoside A	C_33_H_42_O_16_	EtOH	Fruits	Su et al. (2013)
55	Bruceoside B	C_32_H_42_O_16_	EtOH	Seeds	He et al. (2021)
56	Bruceoside C	C_32_H_42_O_16_	EtOH	Seeds	He et al. (2021)
57	Bruceoside D	C_31_H_40_O_16_	MeOH	Seeds	Ohnishi et al. (1995)
58	Bruceoside E	C_31_H_42_O_16_	EtOH	Seeds	He et al. (2021)
59	Bruceoside F	C_35_H_46_O_18_	EtOH	Seeds	Ohnishi et al. (1995)
60	Bruceene	C_20_H_26_O_8_	EtOH	Fruits	Su et al. (2013)
61	Bruceajavanin A	C_34_H_48_NaO_7_	—	Stems	Kitagawa et al. (1994)
62	Bruceajavanin B	C_33_H_49_O_6_	—	Stems	Kitagawa et al. (1994)
63	Brujavanol A	C_20_H_30_O_7_	EtOAc	Roots	Chumkaew and Srisawat, (2017)
64	Brujavanol B	C_20_H_30_O_6_	EtOAc	Roots	Chumkaew and Srisawat, (2017)
65	Brujavanol C	C_21_H_33_O_8_	EtOAc	Stems	Chumkaew et al. (2017)
66	Brujavanol D	C_21_H_33_O_7_	EtOAc	Stems	Chumkaew et al. (2017)
67	Dihydrobruceajavanin A	C_34_H_50_O_7_	—	Stems	Kitagawa et al. (1994)
68	Demethyl-dehydrobrusatol	C_25_H_28_O_11_	EtOH	Seeds	He et al. (2021)
69	Deacetyl-yadanzioside I	C_27_H_36_O_15_	EtOH	Seeds	He et al. (2021)
70	Dehydrobrusatol	C_26_H_30_O_11_	EtOH	Seeds	Liu J. H et al. (2012)
71	Dehydrobruceine B	C_23_H_26_O_11_	EtOH	Seeds	He et al. (2021)
72	Dehydrobruceantinol	C_30_H_36_O_13_	EtOH	Seeds	He et al. (2021)
73	Quassilactone A	C_26_H_35_O_12_	EtOH	Fruits	Su et al. (2020)
74	Quassilactone B	C_26_H_36_O_12_	EtOH	Fruits	Su et al. (2020)
75	3′-hydroxybrucein A	C_26_H_34_O_12_	MeOH	Seeds	Lahrita et al. (2019)
Alkaloids					
76	Bruceolline H	C_13_H_11_NO_3_	MeOH	Stems	Chen et al. (2011)
77	Bruceolline I	C_13_H_13_NO_3_	MeOH	Stems	Chen et al. (2011)
78	Bruceolline J	C_13_H_13_NO_2_	MeOH	Stems	Chen et al. (2011)
79	Bruceolline K	C_19_H_23_NO_7_	MeOH	Stems	Chen et al. (2011)
80	Bruceolline L	C_13_H_15_NO_2_	MeOH	Stems	Chen et al. (2011)
81	Bruceolline M	C_19_H_25_NO_7_	MeOH	Stems	Chen et al. (2011)
82	Bruceolline N	C_19_H_27_NO_9_	MeOH	Stems	Chen et al. (2011)
83	Bruceacanthinoside	C_26_H_28_N_2_NaO_12_	MeOH	Stems	Kitagawa et al. (1994)
Triterpenoids					
84	Brujavanone A	C_34_H_48_NaO_8_	EtOH	Twigs	Dong et al. (2013)
85	Brujavanone B	C_33_H_48_NaO_7_	EtOH	Twigs	Dong et al. (2013)
86	Brujavanone C	C_32_H_46_NaO_6_	EtOH	Twigs	Dong et al. (2013)
87	Brujavanone D	C_33_H_50_NaO_8_	EtOH	Twigs	Dong et al. (2013)
88	Brujavanone E	C_32_H_48_NaO_8_	EtOH	Twigs	Dong et al. (2013)
89	Brujavanone F	C_34_H_52_NaO_8_	EtOH	Twigs	Dong et al. (2013)
90	Brujavanone G	C_33_H_48_NaO_7_	EtOH	Twigs	Dong et al. (2013)
91	Brujavanone H	C_33_H_50_NaO_7_	EtOH	Twigs	Dong et al. (2013)
92	Brujavanone I	C_33_H_52_NaO_7_	EtOH	Twigs	Dong et al. (2013)
93	Brujavanone J	C_33_H_52_NaO_7_	EtOH	Twigs	Dong et al. (2013)
94	Brujavanone K	C_32_H_50_NaO_7_	EtOH	Twigs	Dong et al. (2013)
95	Brujavanone L	C_32_H_48_NaO_6_	EtOH	Twigs	Dong et al. (2013)
96	Brujavanone M	C_39_H_62_NaO_10_	EtOH	Twigs	Dong et al. (2013)
97	Brujavanone N	C_34_H_54_NaO_8_	EtOH	Twigs	Dong et al. (2013)

## Quassinoids

Quassinoids are the major category of anticancer phytochemicals of *B. javanica*. A total of 79 quassinoids have so far been isolated from *B. javanica.* Most of them were reported to have biological activities (Ye et al., 2015). Bruceine A-J, M (1-11) (Polonsky et al., 1967; Duncan and Henderson, 1968; Lee et al., 1979; Zhao et al., 2011; Chen et al., 2013; Su et al., 2013; Li et al., 2021), bruceanic acids E-F, bruceanic acids E methyl ester (12-14) (Liu. et al., 2012), javanicolide A-E, and H (15-20) (Su et al., 2002; Kim et al., 2003; Kim et al., 2004b; Liu. et al., 2012), javanicoside B-G, I-L (21-30) (Kim et al., 2004a; Kim et al., 2004b; He et al., 2021), javanic acids A-B (31-32) (Liu. et al., 2012), yadanzioside A-C, E-G, I,K,L-P (33-45) (Sakaki et al., 1985; Yoshimura et al., 1985; Sakaki et al., 1986; Kim et al., 2004b; Chen et al., 2011; Zhao et al., 2011; Su et al., 2013; Ye et al., 2015; He et al., 2021), yadanziolide C-S (46-47) (Su et al., 2013; He et al., 2021), yadanzigan (48) (Zhan et al., 2020), 20-hydroxyyadanzigan (49) (Zhan et al., 2020), brusatol (50) (Sim et al., 1968), bruceantin (51) (Su et al., 2013), bruceantinol (52) (Kupchan et al., 1975), bruceantinosides A (53) (Zhao et al., 2011), bruceoside A-F (54-59) (Ohnishi et al., 1995; Su et al., 2013; He et al., 2021), bruceene (60) (Su et al., 2013), bruceajavanin A-B (61-62) (Kitagawa et al., 1994), brujavanol A-D (63-66) (Chumkaew et al., 2017; Chumkaew and Srisawat, 2017), dihydrobruceajavanin A 67) (Kitagawa et al., 1994), demethyl-dehydrobrusatol (68) (He et al., 2021), deacetyl-yadanzioside I (69) (He et al., 2021), dehydrobrusatol (70) (Liu. et al., 2012), dehydrobruceine B (71) (He et al., 2021), dehydrobruceantinol (72) (He et al., 2021), quassilactone A-B (73-74) (Su et al., 2020) and 3′-hydroxybrucein A (75) (Lahrita et al., 2019) are the quassinoids isolated from *B. javanica*. Among which, brusatol (50) and bruceine D (4) are two important bioactive compounds of *B. javanica*. Our previously studies have shown that brusatol (50) and bruceine D (4) exhibited potent cytotoxicity on several cell lines of pancreatic cancer, with IC_50_ values of 0.36 µM (PANC-1)/0.10 µM (SW 1990) for brusatol and 2.53 µM (PANC-1)/5.21 µM (SW 1990) for bruceine D, respectively (Lau et al., 2009; Zhao et al., 2011; Lu et al., 2017). Apart from brusatol and bruceine D, several constituents such as bruceantin (51) and bruceantinol (52) were reported to exhibit potent antineoplastic activity. Furthermore, four quassinoid glucosides, i.e., javanicosides I, J, K and L (28-31), isolated from *B. javanica*, showed moderate cytotoxic activity on P-388 murine leukemia cells, with IC_50_ values of 7.5, 2.3, 1.6 and 2.9 μg/ml, respectively (Kim et al., 2004a). Brujavanol A (63) and brujavanol B (64) exhibited significant cytotoxicity against human oral cavity cancer (KB) cells, with IC_50_ values of 1.3 and 2.36 μg/ml, respectively (Chumkaew and Srisawat, 2017).

## Alkaloids

Alkaloids are important secondary metabolites of this plant and play an important role in the organism’s natural defense (Heinrich et al., 2021). Until now, 8 alkaloids, *viz*., bruceolline H-N (76-82) (Kitagawa et al., 1994; Chen et al., 2011) have been isolated from the stems of *B. javanica* and their chemical structures elucidated*.* However, no study has investigated the biological properties of these alkaloids so far.

## Triterpenoids

Triterpenoids represent another relatively smaller class of compounds from *B. javanica.* Fourteen apotirucallane-type triterpenoids, namely brujavanone A-N, (84-97) were isolated from the twigs of *B. javanica* (Dong et al., 2013).

## Pharmacological Properties of the *B. javanica*-Derived Chemicals

**FIGURE 2:**
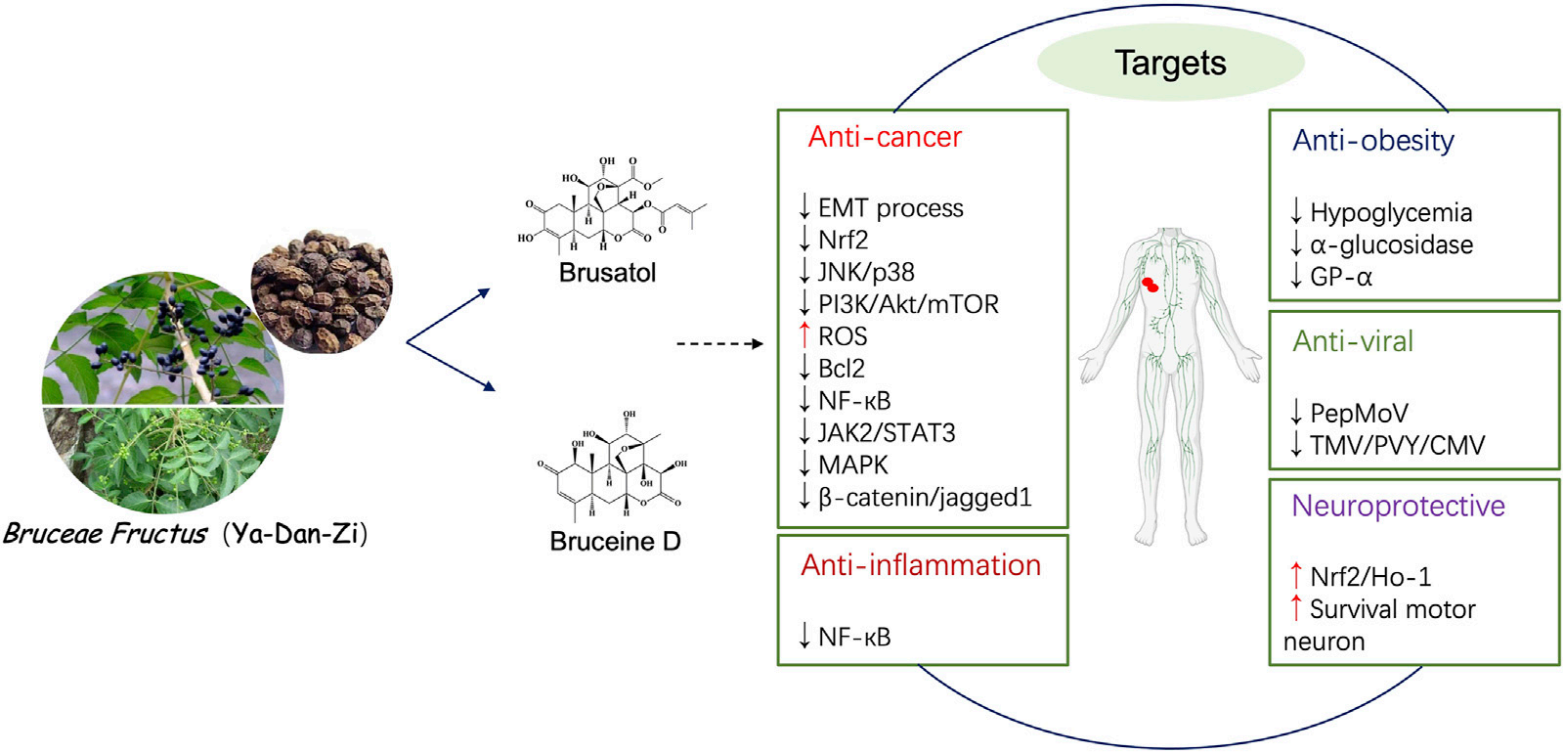
Schematic presentation of the pharmacological activities of brusatol and bruceine D, the two main constituents of *B. javanica*.

The fruits of *B. javanica* are commonly used in clinical practice to treat various diseases. The chemical compounds isolated from *B. javanica* possess a wide range of biological activities such as anti-tumor, anti-diabetic and neuroprotective actions. The typical and representative pharmacological effects of *B. javanica*-derived chemical compounds are summarized in Table 2 and Figure 2 below.

**TABLE 2:** Pharmacological activities of the bioactive compounds derived from *B. javanica.*

Pharmacological activity	Compounds	Cancer types	Cells	Mechanism/Effects	Ref.
Anti-cancer	Brusatol	Pancreatic cancer	PANC-1/Capan-2	Suppresses the EMT process	Lu et al. (2017)
			PANC-1/BXPC-3	Suppresses the Nrf2 pathway	Xiang et al. (2018)
			PANC-1/PATU-8988	Inhibits JNK/p38/MAPK/NF-κb/Stat3/Bcl-2 signaling pathway	Xiang et al. (2017)
		NSCLC	A549/H1229	Promotes ROS production and enhances DNA damage	Sun et al. (2016)
			A549/H1650/PC9/HCC827	ROS-mediates mitochondrial-dependent pathway and inhibits the Nrf2-mediate antioxidant response	Xie et al. (2021)
		Breast cancer	BT-474/SK-BR-3	Inhibits Nrf2/HO-1 and HER2-AKT/ERK1/2 Pathways	Yang et al. (2020b)
			MDA-MB-231	Inhibits the EMT process and increases ROS production	Chandrasekaran et al. (2021)
		HCC	HCCLM3	Affects EMT process through modulation of STAT3 activation pathway	Lee et al. (2020)
			Bel7404/Huh7/Hep3B	Induces autophagy *via* the PI3K/Akt/mTOR pathway	Ye et al. (2018)
		CRC	CT-26	Decreases the expression of procaspase-3 and procaspase-9, and upregulation of the B-cell lymphoma 2 (Bcl-2)-associated X protein/Bcl-2 ratio	Chen et al. (2018)
			RKO/HCT116	Inhibits the c-Myc expression and increases HIF-1*α* degradation	(Lu et al., 2016; Oh et al., 2017)
			CRC orthotopic model	Nrf2 inhibitor	Evans et al. (2018)
		NPC	CNE-1/CNE-2/5-8F/6-10B	Suppresses the Akt/mTOR signaling pathway	Guo et al. (2020)
		Pituitary adenoma	GH3/MMQ	Increases production of ROS and inhibits the phosphorylation of 4EBP1 and S6K1	Wu et al. (2021)
		Gastric cancer	SGC-7901	Inhibits PI3K/Akt/NF-кB pathway	Chen et al. (2021)
		Head and Neck Squamous	UMSCC 47	Regulates STAT3 signaling	Lee et al. (2019)
		Melanoma	A375	Inhibits the Nrf2 signaling	Wang et al. (2018)
		Laryngeal cancer	Hep-2	Abrogates JAK2/STAT3 signaling mediated EMT process	Zhou et al. (2021)
	Bruceine D	PanCa	PANC-1	Mediates p38-mitogen-activated protein kinase and NF-κB signaling pathways	(Lau et al., 2009; Lau et al., 2010)
			Capan2	Inhibits mitochondrial pathway	Liu L et al. (2012)
		NSCLC	A549/H1650/HCC827	Modulates the ROS-mitochondrial-mediated death signaling	Xie et al. (2019)
			A549/NC-H292	Regulates the ROS/MAPK signaling pathway	Fan et al. (2020)
			A549/H460	Downregulates JNK pathway	Tan et al. (2019)
		Breast cancer	MDA-MB-231	Downregulates the expression of PI3K and reduces AKT phosphorylation	Luo et al. (2020)
			MDA-MB-231/MCF-7	Modulates MAPK signaling cascade	Mohan et al. (2021)
		HCC	Huh7/Hep3B	Downregulates *β*-catenin/jagged1 pathway	Cheng et al. (2017)
			PLC/Hep3B	Downregulates the expression of miR-95	Xiao et al. (2014)
		OSCs	MNNG-HOS/U-2OS	Inhibits STAT3 signaling pathway	Wang et al. (2019)
		CML	K562	Inhibits phosphorylation of AKT and ERK	Zhang et al. (2016)
		Gastric cancer	HGC27/MKN45/SGC7901	Downregulates the LINC01667/miR-138-5p/Cyclin E1 axis	Li et al. (2020)
	Bruceine A	PanCa	MIA PaCa-2	Activates p38*α* MAPK signaling	Lu et al. (2021)
			MIA PaCa-2	Inhibits PFKFB4/GSK3*β* signaling	Zhang P. F et al. (2021)
	Bruceantin	Prostate caner	22RV1/C4-2B	Inhibits HSP90 chaperone function	Moon et al. (2021)
Anti-obesity	Bruceine D/E	—	—	Exhibits hypoglycemia effect	NoorShahida et al. (2009)
	Bruceine D/E	T2D	—	Inhibits *α*-glucosidase and GP-*α*	Ablat et al. (2017)
Anti-viral	Brusatol	TMV	—	—	Yan et al. (2010)
		PepMoV	—	Against PepMoV	Ryu et al. (2017)
	Bruceine D	—		Inhibits TMV, PVY and CMV	Shen et al. (2008)
	Bruceine D	ZIKV	—	Inhibits ZIKV infection at a post-entry stage	Zhang P. F et al. (2021)
Neuroprotective	Brusatol	—	U-251	Induces Nrf2/HO-1 pathway	Liu et al. (2019)
	Bruceine D	Parkinson’s disease	—	Activates Nrf2 expression	Yang et al. (2020a)
		Spinal muscular atrophy	—	Corrects the survival motor neuron 2 splicing defect	Baek et al. (2019)
Anti-inflammatory	Bruceine D	Ulcerative colitis	—	Suppresses NF-κB pathway	Dou et al. (2018)

### Anti-Cancer Effects

The anti-cancer activity is one of the most intensively studied biological effects for *B. javanica*. Many *in vitro* and *in vivo* studies have demonstrated the significant anti-cancer effects of *B. javanica*-derived chemicals in various types of cancer, such as pancreatic cancer, lung cancer, breast cancer, hepatocellular carcinoma (HCC), colorectal cancer (CRC), gastric cancer and leukemia. The molecular mechanisms of the antitumor activity of the isolated compounds from *B. javanica* are shown in Table 2 and Figure 2.

### Anti-Pancreatic Cancer

Brusatol, one of the major compounds isolated from *B. javanica*, shows various anti-cancer effects. Our previous studies demonstrated that brusatol (50) could synergistically enhance the anti-pancreatic cancer effects of gemcitabine/5-fluorouracil, and its underlying molecular mechanism was associated with the suppression of epithelial-mesenchymal transition (EMT) process, in which epithelial cells lose their cell polarity and cell adhesion, and gain migratory and invasive properties to become mesenchymal stem cells. The EMT process is very intimately associated with initiation of metastasis in cancer progression (Lu et al., 2017). Brusatol was also found to potentiate the gemcitabine-induced growth inhibition and apoptosis, and enhance the chemotherapeutic efficacy of gemcitabine both in human pancreatic cancer cells and PANC-1 xenografts *via* suppressing the Nrf2 pathway (Xiang et al., 2018). Furthermore, brusatol can induce cell apoptosis and inhibit cell growth in pancreatic cancer through JNK/p38/MAPK/NF-κb/Stat3/Bcl-2 signaling pathway (Xiang et al., 2017). In other studies, NF-*κ*B and signal transducer and activator of transcription 3 (Stat3) were found to be activated in pancreatic cancer, and bruceine D (4) could inhibit cell proliferation and induce cell apoptosis *via* attenuating the activation of the redox-sensitive p38-MAPK pathway (Lau et al., 2009) and suppressing NF-*κ*B anti-apoptotic activity (Lau et al., 2010). Moreover, bruceine D (4) was able to induce cytotoxicity in Capan-2 cells through induction of cellular apoptosis involving the mitochondrial pathway (Liu j. H et al., 2012). A recent study showed that bruceine A (1), another quassinoidal compound, displayed potent anti-proliferative activity in *in vitro* and *in vivo* pancreatic cancer models through directly activating p38*α* MAPK signaling (Lu et al., 2021). Other investigation also showed that bruceine A (1) could induce cell growth inhibition and apoptosis *via* 6-phosphofructo-2-kinase/fructose-2,6-bisphosphatase 4 (PFKFB4)/GSK3*β* signaling in pancreatic cancer cells (Zhang. et al., 2021). It should be noted that PFKFB4 is a bifunctional metabolic enzyme that stimulates glycolysis, while GSK3*β* is a serine/threonine kinase and a key regulator of glycogen synthesis and energy homeostasis.

### Anti-Lung Cancer

Brusatol (50) could enhance the radiosensitivity of A549 lung cancer cells by promoting reactive oxygen species (ROS) production and elevating DNA damage (Sun et al., 2016). Additionally, brusatol (54) markedly inhibited the growth, clonogenic capability and migratory ability of non-small-cell lung cancer (NSCLC) cells through mediating ROS-mitochondrial-dependent pathway and inhibiting the Nrf2-mediated antioxidant response, which is the key transcription factor that regulates the antioxidant response (Xie et al., 2021). Meanwhile, bruceine D (4) could induce NSCLC apoptosis *via* modulating ROS-mediated death signaling and inhibiting the expression of the anti-apoptotic proteins Bcl-2, Bcl-xL and X-linked inhibitor of apoptosis, while it increased the expression levels of apoptotic proteins Bax and Bak, and suppressed the expression of pro-caspase-3 and pro-caspase-8 (Xie et al., 2019). Moreover, bruceine D (4) could induce mitochondria-dependent cell apoptosis, inhibit cell proliferation and suppress the growth of lung cancer xenografts *via* regulating the ROS/MAPK signaling pathway (Fan et al., 2020). It has also been shown that bruceine D (4) was able to inhibit the proliferation and increase the apoptosis of A549 and H460 NSCLC cells through downregulating the JNK pathway (Tan et al., 2019).

### Anti-Breast Cancer

Brusatol (50), a well-established Nrf2 inhibitor, could enhance the anticancer activity of HER2-targeted trastuzumab in breast cancer by inhibiting Nrf2/HO-1 and HER2-AKT/ERK1/2 pathways (Yang et al., 2020b). Further mechanism studies demonstrated that brusatol (50) inhibited cell metastasis, induced cell apoptosis and enhanced the chemotherapeutic efficacy of paclitaxel on triple-negative breast cancer through inhibition of EMT process and attenuation of the ROS production (Chandrasekaran et al., 2021). In another study, bruceine D (4) was found to inhibit cell viability, migration and invasion of triple-negative breast cancer MDA-MB-231 cells in a dose-dependent manner through suppression of PI3K/AKT pathway (Luo et al., 2020). A recent report has also shown that bruceine D (4) can enhance the apoptosis and interfere with cellular invasion by regulating MAPK signaling pathway in MDA-MB-231 and MCF-7 breast cancer cells (Mohan et al., 2021).

### Anti-HCC

Brusatol (50) was found to attenuate STAT3-driven metastasis in HCC by altering the level of EMT-related proteins (Lee et al., 2020). Brusatol (50) effectively inhibited proliferation and induced apoptosis to inhibit tumor invasion and migration in HCC *via* modulating the PI3K/Akt/mTOR pathway, which plays an important role in the regulation of signal transduction and biological process such as cell apoptosis, proliferation, metabolism and angiogenesis (Ye et al., 2018). Bruceine D (4) was able to inhibit the proliferation, promote apoptosis of HCC cells and enhance the inhibitory efficacy of sorafenib in HCC *via* downregulating the expression of *β*-catenin and jagged 1 (Cheng et al., 2017). It has been shown in another study that bruceine D (4) exerts anti-cancer activity against HCC through modulation of miR-95 expression (Xiao et al., 2014).

### Other Cancer Types

A recent study showed that brusatol (50) was able to produce a synergistic antitumor effect in CRC when combined with cisplatin (Chen et al., 2018). Hypoxia-inducible factor-1 (HIF-1*α*) is a dimeric protein complex that is involved in the homeostatic process and it can increase vascularization in hypoxic areas such as tumors. Brusatol (50) could also induce the cell death of CRC by inhibiting c-Myc expression and increasing HIF-1*α* degradation (Oh et al., 2017) (Lu et al., 2016). Furthermore, brusatol (50), as a Nrf2 inhibitor, could effectively abrogate CRC tumor growth both in subcutaneously and orthotopically-allografted mice (Evans et al., 2018). Another study has reported that brusatol (50) exerts anti-proliferative activity by inducing the mitochondrial apoptosis and cell cycle arrest against nasopharyngeal carcinoma (NPC), and significantly inhibits the growth of NPC CNE-1 xenografts with no overt toxicity through suppression of Akt/mTOR signaling pathway (Guo et al., 2020). Bruceantin (51) could efficiently suppress tumor growth and metastasis of castration-resistant prostate cancer cells and overcome resistance caused by aberrant full-length androgen receptor (AR-FL)/AR-V7 signaling *via* targeting HSP90 expression (Moon et al., 2021).

Cabergoline (CAB) is the first choice for the treatment of prolactinoma, which is the most common subtype of pituitary adenoma. Treatment with brusatol (50) could lead to the inhibition of tumor growth and increase the efficacy of CAB against pituitary adenoma through inducing the overproduction of ROS and inhibiting the phosphorylation of 4EBP1 and S6K1 (Wu et al., 2021). Osteosarcoma stem cells (OSCs) are a potential cause of tumor metastasis, relapse, and chemotherapy resistance. It was reported that bruceine D (4) exerted significant anti-osteosarcoma activity *via* inhibiting cell proliferation and migration, inducing cell cycle arrest and promoting apoptosis in osteosarcoma cells. Besides, bruceine D (4) could also suppress the sphere-forming and self-renewal ability of OSCs. Mechanistically, the inhibitory role of bruceine D (4) on osteosarcoma cell growth and migration was partially executed *via* inhibition of STAT3 signaling pathway (Wang et al., 2019). Chronic myeloid leukemia (CML), an acquired malignant myeloproliferative disorder of hematopoietic stem cells, is one of the three most common forms of leukemia. A study revealed that bruceine D (4) could induce apoptosis and inhibit tumor growth in human CML K562 cells *via* regulating mitochondrial pathway, which is the main energy metabolism pathway and plays a critical role in pituitary adenomas (Zhang et al., 2016). Additionally, bruceine D (4) could also inhibit cell proliferation and induce cell cycle arrest at S phase and enhance the chemosensitivity of doxorubicin on gastric cancer cells by downregulating the expression of a long non-coding RNA LINC01667/miR-138-5p/Cyclin E1 axis (Li et al., 2020). Brusatol was able to induce apoptosis of human gastric cancer SGC-7901 cells *via* modulating of PI3K/Akt/NF-кB pathway (Chen et al., 2021). Moreover, brusatol (50) also showed anti-cancer activity in head and neck squamous cell carcinoma (Lee et al., 2019), melanoma (Wang et al., 2018) and laryngeal cancer (Zhou et al., 2021) through inhibiting STAT3 and the Nrf2 signaling pathways and abrogating JAK2/STAT3 signaling-mediated EMT process, respectively. Evidence has been accumulated that the value of brusatol as a new strategy for cancer treatment as it spefifically targets Nrf2 defensive mechanism. The study on anti-cancer action of brusatol may open a new pathway for future drug development and clinical translation (Cai et al., 2019).

## Anti-Diabetic Effect

Obesity, defined as an excess of white adipose tissue, is related to a higher risk of developing diabetes and cardiovascular disease (Marques et al., 1998). *B. javanica* has been shown to possess anti-diabetic activity, and ethnopharmacological study showed that the Fructus Bruceae is recommended by traditional practitioners for the treatment of diabetes mellitus. Bruceine D (4) and bruceine E (5) were found to exhibit hypoglycemic effect in normoglycemic and streptozotozin (STZ)-induced diabetic rats. Normoglycemic mice administered with 1 mg/kg of bruceine D and bruceine E showed significant reduction in blood glucose concentration by 40.07 ± 11.45% and 48.82 ± 13.34%, respectively. Administration with bruceine D and bruceine E caused significant blood glucose concentration reduction by 73.57 ± 13.64% and 87.99 ± 2.91%, respectively, in STZ-induced diabetic rats (NoorShahida et al., 2009; Man and Choo, 2017). Another study also revealed that bruceine D (4) and bruceine E (5) had potential therapeutic value for the treatment of type 2 diabetes *via* acting as *α*-glucosidase and glycogen phosphorylase *α* (GP-*α*) inhibitors, thereby improving hepatic glucose and carbohydrate metabolism, inhibiting oxidative stress, and preventing inflammation in type 2 diabetic (T2D) rats (Ablat et al., 2017).

## Anti-Viral Effects

Several studies indicated that the compounds isolated from *B. javanica* exhibited strong inhibitory effects against various plant viruses, especially tobacco mosaic virus (TMV) and pepper mottle virus (PepMoV). PepMoV belongs to the genus *Potyvirus* in the family *Potyviridae* and is composed of a filamentous particle with a positive single-stranded RNA genome. It predominantly infects *Capsicum species*. A recent study demonstrated that brusatol (50) exerted significant antiviral activities against TMV (Yan et al., 2010) and PepMoV (Ryu et al., 2017) in the host plants. It was reported that bruceine D (4) could also possess anti-phytoviral activity against TMV, potato virus Y (PVY) and cucumber mosaic virus (CMV) (Shen et al., 2008). Zika virus (ZIKV) is associated with severe birth defects and Guillain-Barré syndrome; however, no effective vaccines or therapies are currently available to conquer ZIKV infection. Several plant-derived compounds have been screened for their ability to block ZIKV infection. Results showed that bruceine D (4) significantly inhibited the ZIKV, with the IC_50_ less than 1 μM (Zhang J. W et al., 2021).

## Neuroprotective Effect

Chemical compounds derived from *B. javanica* were also found to exert neuroprotective effects. Recent research using glioma U-251 cells to study the biological processes of amyloid-*β* (A*β*)-induced neurotoxicity demonstrated that brusatol (50) could effectively ameliorate cell injury and inhibit A*β*-induced neurotoxicity *via* inducing Nrf2/HO-1 and PI3K/AKT/mTOR (Liu et al., 2019). In addition, bruceine D (4) was reported to markedly improve the loss of dopaminergic neurons in the substantia nigra pars compacta (SNpc) and alleviate neuroinflammation through reducing the glial activation in 1-methyl-4-phenyl-1,2,3,6-tetrahydropyridine (MPTP)-induced mouse model of Parkinson’s disease. Moreover, oxidative stress in MPTP mice was attenuated after bruceine D treatment, and the mechanism of action was associated with improving the Nrf2 activation (Yang et al., 2020a). Furthermore, bruceine D (4) could improve the spinal muscular atrophy (SMA) through enhancing the survival of motor neuron 2 splicing defect contributed by a reduction in the expression of heterogeneous nuclear ribonucleoprotein A1 (hnRNP A1) (Baek et al., 2019). The findings suggest a good potential for developing bruceine D into a plant-derived SMA treatment.

## Others

Besides the pharmacological activities alluded to above, some scattered researches have reported additional pharmacological effects of *B. javanica*-derived compounds such as anti-bacterial and anti-inflammatory actions*.* Bruceine D (4) was found to effectively alleviate colonic inflammation in trinitrobenzenesulfonic acid-induced ulcerative colitis in rats by suppressing NF-κB pathway (Dou et al., 2018).

## Clinical Studies

To date, no clinical data is available to support the use of *B. javanica*-derived chemical compounds for the treatment of cancer*.* However, two patented products of BJO, i.e., BJO emulsion injection (BJOE) and BJO soft capsule, have been extensively used in China as an adjuvant therapy to conventional chemotherapy for the treatment of malignant tumors. Clinical investigations on BJOE revealed its potential to reduce the postoperative adverse reactions, improve the quality of life and enhance the total curative rate of the cancer patients (Li et al., 2021). A study conducted on 1399 patients with lung cancer showed that BJOE treatment resulted in an improved treatment effect [RR 1.36, 95%CI (1.23,1.51), *p* < 0.0001], and patient’s quality of life [RR 2.11, 95% CI (1.66,2.67), *p* < 0.0001] and improved side effects compared with DP (Docetaxel combined with Cisplatin) regimen (Mei et al., 2021). In another randomized controlled trial which was carried out to examine the efficacy and safety of BJOE in patients with brain metastasis tumor, the results showed that BJOE significantly increased the patients’ disease response rate, protected immune function, improved quality of life, as well as dramatically lowered the incidence of rest of bone marrow and gastroenteric reaction (Zhang et al., 2017). Moreover, a meta-analysis was conducted to determine the efficacy and safety of BJOE combined with transcatheter arterial chemoembolization (TACE) in treating moderate or advanced primary liver cancer. The results showed that BJOE (30 ml/d) combined with TACE significantly increased overall efficacy, 2-year survival rate, quality of life, and decreased the incidence of leukopenia (*p* < 0.05) when compared with TACE alone (Liu et al., 2017).

The results of the above clinical studies indicate that Fructus Bruceae*,* which is one of the most potent Chinese herbs with good antitumor activity, is a promising naturally occurring agent to be developed into anti-cancer treatment for patients with solid tumors in future.

## Discussion and Future Perspective

As an important historical herbal medicine, Fructus Bruceae has been used in a variety of clinical application. Owing to its diverse bioactive properties, *B. javanica* has attracted much attention of the research community in recent decades. The present review aims to achieve in systematically and comprehensively summarizing the phytochemical compounds and the pharmacological activities of *B. javanica.* More than a hundred chemical compounds have been isolated and identified from different parts of this plant, and the main chemical classes of these isolates include quassinoids, alkaloids and triterpenoids. Through a comprehensive analysis, we found that brusatol and bruceine D are the major active compounds of *B. javanica* as they possess many pharmacological activities, including anti-cancer, anti-diabetic, antiviral, anti-inflammatory, and anti-bacterial properties.

Several limitations are also noted with current status of research on *B. javanica*, which call for further research efforts. These include 1) although many chemical constituents have been isolated and identified from this plant*,* only a handful of these components, such as brusatol and bruceine D, have been subjected to pharmacological evaluations. Hence, in-depth pharmacological studies, especially concentrating on the elucidation of the molecular mechanism of the bioactive compounds, shall undoubtedly be the focus of future investigation. The wide range of pharmacological properties possessed by *B. javanica* could present us with novel pathway for the disease management. 2) Toxicological studies are essential to understand the safety of herbal drugs; however, the data on toxicological aspects of *B. javanica* still remain scarced. Although research has suggested that many parts of this plant possess little or no toxicity, bruceine D has been shown to have some adverse reactions (Fan et al., 2020). Therefore, toxicity and safety assessment on bruceine D and other active constituents needs to be conducted to fully unravel the safety profile of this medicinal drug and its bioactive constituents. 3) Besides, many chemical constituents derived from this plant have poor solubility which could hinder the clinical application of these chemicals (Zou et al., 2017). How to improve the solubility and bioavailability certainly warrants exploration in the future.

Finally, given that BJO emulsion injection and BJO soft capsule have been demonstrated to possess good clinical efficacy in the treatment of some solid tumors, and their use in clinical practice has been for a long time, it is reasonable to hypothesize that the major chemical constituents such as brusatol and bruceine D may possess even more potent anti-cancer effects if used in clinical setting. In this regard, we believe that conducting clinical trials to evaluate the efficacy and safety of these two major *B. javanica*-chemicals for solid tumors such as pancreatic cancer and liver cancer shall be a worthy scientific pursuit in the near future.
